# Unusual presentation of bilateral tuberculous otomastoiditis with tuberculous spondylitis in a 14-year-old child: A case report

**DOI:** 10.1016/j.radcr.2024.03.060

**Published:** 2024-04-16

**Authors:** Reyhan E. Yunus, Ayu A. Sriyana

**Affiliations:** a Department of Radiology, Dr. Cipto Mangunkusumo General Hospital-Faculty of Medicine University of Indonesia, Jakarta, Indonesia; b Department of Otorhinolaryngology, Head and Neck Surgery, Dr. Cipto Mangunkusumo General Hospital-Faculty of Medicine University of Indonesia, Jakarta, Indonesia

**Keywords:** Children, Mastoiditis, Otitis media, Spondylitis, Tuberculosis

## Abstract

Tuberculous otomastoiditis, a rare manifestation of tuberculosis in the head and neck region, poses diagnostic and therapeutic challenges due to its non-specific clinical features and potential debilitating complications. While typically arising from direct spread from adjacent organs, the coexistence of tuberculous otomastoiditis and cervical spondylitis is rarely reported. We present the case of a 14-year-old male with a 3-month history of painless bilateral ear discharge resistant to antibiotic therapy. The clinical and radiological findings raised suspicions of tuberculous otomastoiditis and spondylitis, which was later confirmed by histopathological examination despite negative microbiological cultures. This case underscores the significance of considering tuberculosis in conditions involving multiple organs, especially when persistent extensive damage is observed despite optimal initial treatments.

## Introduction

Tuberculous otomastoiditis is a rare condition resulting from *Mycobacterium tuberculosis* infection in the middle ear and mastoid air cells accounting for approximately 0.05%-0.9% of chronic middle ear infections [1]. While the incidence of tuberculous otomastoiditis has declined in recent decades [2], it remains a crucial differential diagnosis in tuberculosis-endemic countries such as Indonesia, reporting around 354 cases per 100,000 populations [3]. This disease poses a diagnostic challenge due to its nonspecific clinical features, yet carries the potential risk for serious complications leading to significant morbidity and mortality [1]. While tuberculosis infection may involve multiple organs [4], the simultaneous occurrence of tuberculous otomastoiditis and spondylitis has been rarely described. In this case report, we described a rare instance of coincident tuberculous otomastoiditis and tuberculous spondylitis in a young male.

## Case report

**Fig. 1:**
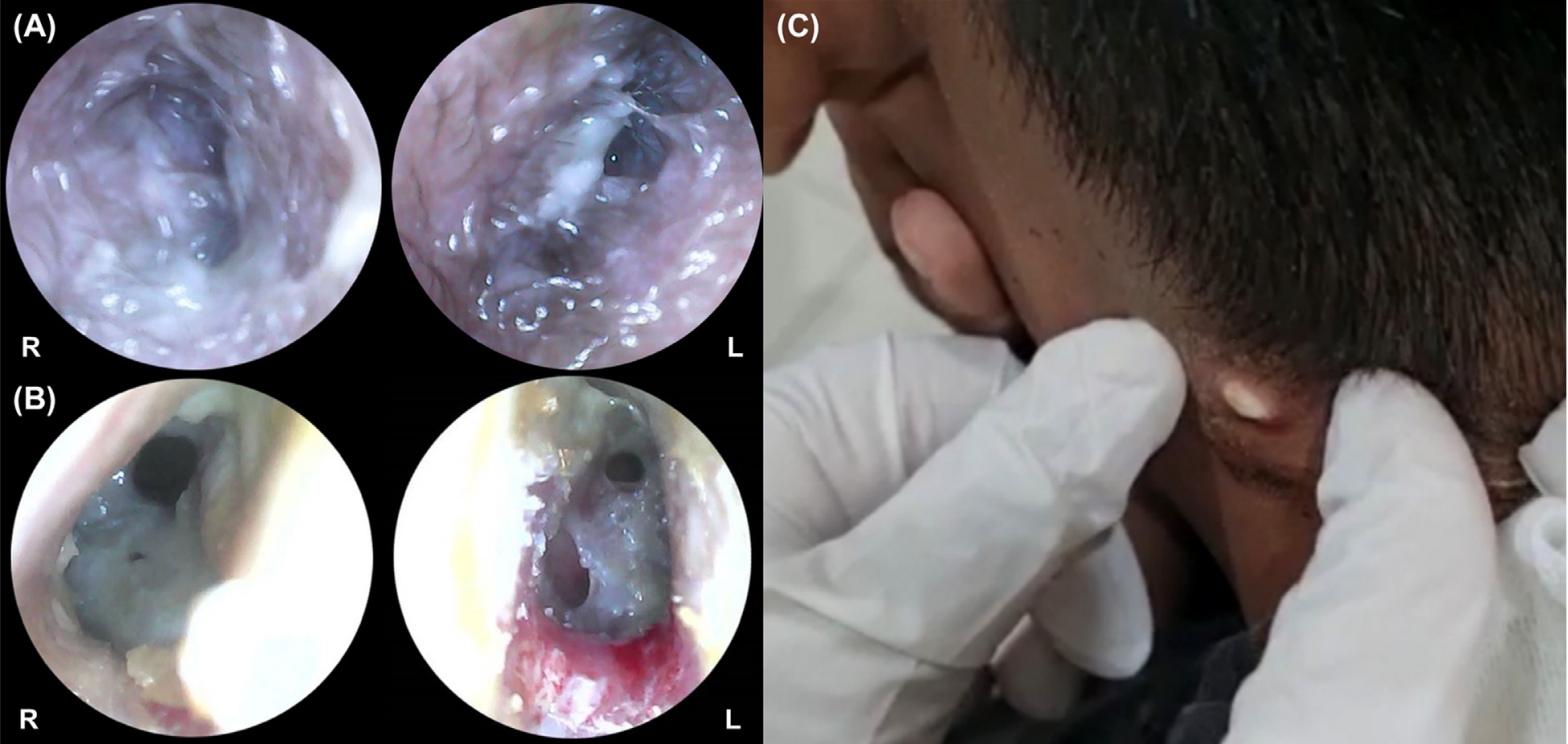
Clinical findings before (A and C) and after (B) the initiation of anti-tuberculosis drugs, illustrating (A) ear discharge and shagging of the external ear canal in both ears, (B) central perforation of the right tympanic membrane and multiple perforations of the left tympanic membrane, and (C) left neck fistula with slough formation.

**Fig. 2:**
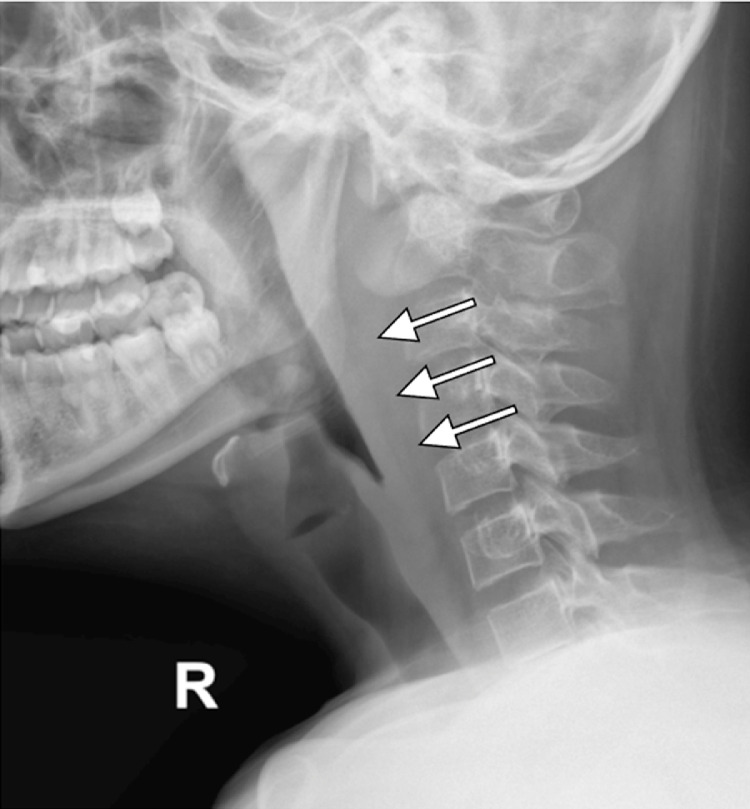
Lateral cervical radiography showing thickening of retropharyngeal and prevertebral soft tissues at the level of the craniocervical junction to the C5 vertebrae (white arrows).

**Fig. 3:**
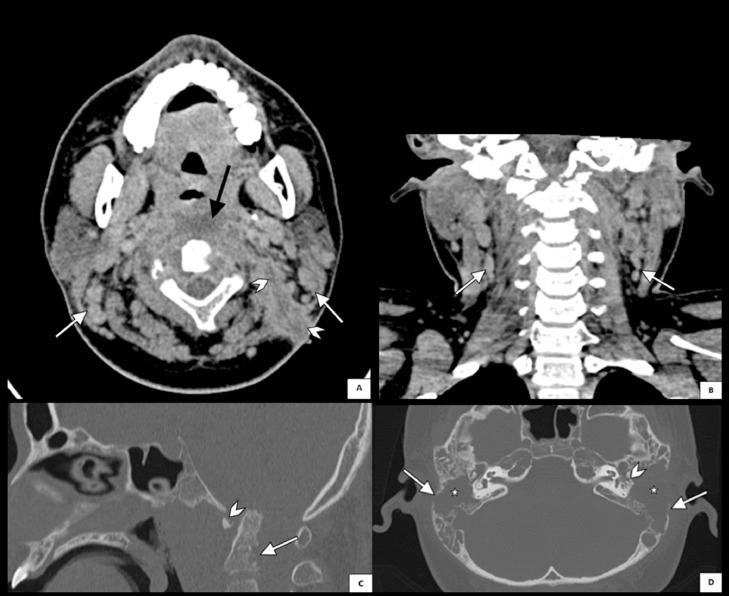
(A) Axial computed tomography (CT) scan of the brain shows an abscess with prevertebral involvement (black arrow) extending to the retropharyngeal and lateral wall with fistula formation (arrowheads), and enlargement of multiple bilateral level II-III cervical lymph nodes (white arrows). (B) Coronal CT scan of the neck also reveals enlargement of bilateral lymph nodes surrounding the neck (white arrows). (C) Sagittal CT scan of the temporal bone with bone window displays erosion of the clivus (arrowhead) and the C1-C2 vertebrae (white arrow). (D) High-resolution CT scan of the mastoid shows destruction of bilateral mastoid air cells opacity (stars) and erosion of the temporal bone with bony sequester and irregular borders (white arrows), with more prominent destruction of the left ossicles (arrowhead) suggesting tuberculous otomastoiditis.

**Fig. 4:**
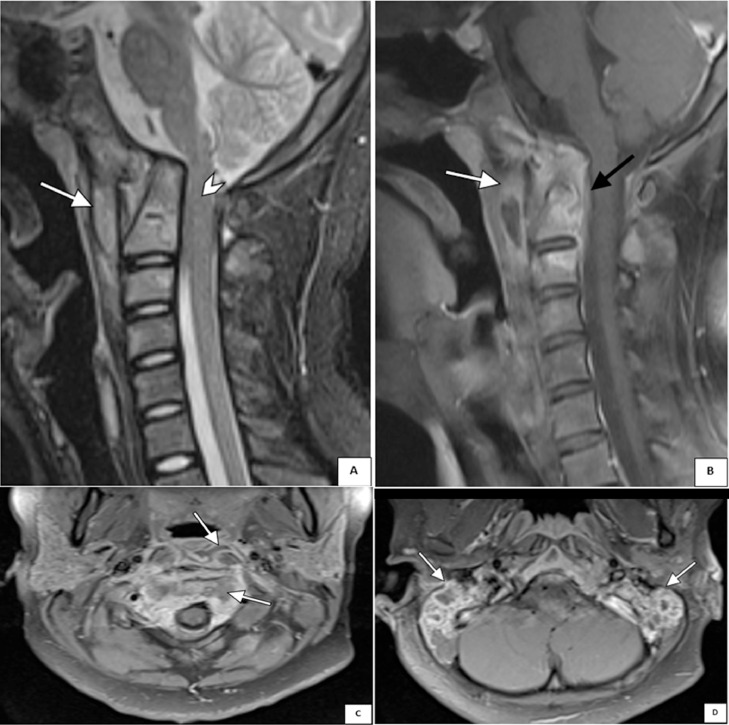
(A) Sagittal T2-weighted magnetic resonance image (MRI) of the cervical spine shows an abscess in the retropharyngeal space (white arrow) and stenosis of the spinal cord at the craniocervical junction level (arrowhead). (B) Sagittal contrast T1-weighted MRI image demonstrates a paravertebral abscess with posterior epidural involvement (white arrow), consistent with spondylitis (black arrow). (C) Axial contrast T1-weighted MRI image reveals an abscess in the retropharyngeal space extending to the spinal canal. (D) Axial contrast T2-weighted MRI image of the mastoid shows heterogeneous intensity of bilateral abscess.

A 14-year-old male presented with persistent bilateral ear discharge over the preceding 3 months. At the time of presentation, the patient reported hearing loss and neck pain but denied experiencing ear pain, discomfort, vertigo, or facial palsy. Two months earlier, an abscess had developed on the lateral side of his neck. The patient was initially diagnosed with otitis media with a neck abscess at a previous hospital, but was then referred to our center due to unresponsiveness to antibiotic therapy. On physical examination, notable findings included purulent discharge from both ears, ear canal edema (Fig. 1A), and central perforation of the right tympanic membrane and multiple perforations of the left tympanic membrane (Fig. 1B). Additionally, a fistula with slough formation was found on the left side of the neck (Fig. 1C). Cervical X-ray revealed thickening of retropharyngeal and prevertebral soft tissues, extending from the craniocervical junction to the C5 vertebra (Fig. 2). Axial computed tomography (CT) scan of the brain showed that the paravertebral abscess extended to the retropharyngeal and lateral wall, with fistula formation (Fig. 3A); and together with coronal CT scan of the neck, showed bilateral enlargement of level II and III cervical lymph nodes (Figs. 3A-B). High-resolution CT scan of the mastoid and temporal bone showed mastoiditis with lateral wall destruction and abscess within the prevertebral, atlantooccipital joint, and spinal spaces (Figs. 3C-D). In addition, magnetic resonance imaging (MRI) delineated osteomyelitis in bilateral occipital condyles extending to the C1 and C2 vertebrae, septic arthritis in the atlantooccipital joint, and spinal meningitis (Fig. 4). There was no evidence of intracranial extension.

The collective symptoms, radiological findings, and the presence of left neck fistula raised suspicions of tuberculosis infection. Consequently, a thorough examination for tuberculosis, encompassing both microbiological and histopathological assessments, was conducted. Microbiological examination of pus from the ear discharge, left neck abscess, and paravertebral abscess returned negative results for acid-fast bacilli, GeneXpert MTB/RIF, and *Mycobacterium tuberculosis* culture. Histopathological findings revealed chronic granulomatous inflammation with caseous necrosis and datia Langhans cells suggestive of tuberculosis infection.

Based on the findings, the patient was diagnosed with tuberculous otomastoiditis, tuberculous cervical spondylitis, and a paravertebral abscess. Subsequently, the patient was admitted to the hospital, immobilized with a neck collar, and initiated on anti-tuberculosis treatment. Two weeks later, spine debridement surgery was performed, while ear surgery was deferred until the completion of tuberculosis treatment and improvement of the paravertebral abscess. The patient exhibited significant improvement in signs and symptoms following anti-tuberculosis therapy. Subsequent recovery from both treatments and surgeries was uneventful.

## Discussion

The present case highlighted the findings of a rare occurrence of coexistent bilateral otomastoiditis and cervical spondylitis caused by tuberculosis infection. While extrapulmonary tuberculosis accounts for approximately 20%-30% of overt tuberculosis infections [5,6], the most common form of extrapulmonary tuberculosis is lymphatic tuberculosis, followed by pleural, genitourinary, and osteoarticular tuberculosis [5]. Tuberculous otitis media is a very rare entity accounting for less than 1% of extrapulmonary tuberculosis [1]; while although tuberculous spondylitis accounts for about 11% of extrapulmonary tuberculosis, cervical spondylitis only contributes to less than 1% of tuberculous spondylitis [7]. However, although uncommon, these conditions are among the most dreadful forms of extrapulmonary tuberculosis, especially considering that cervical tuberculous spondylitis may cause total paralysis [7] and tuberculous otomastoiditis may cause meningitis through the destruction of mastoid air cells in the middle ear [1].

In our case, tuberculous otomastoiditis was suspected as the patient's symptoms did not improve with antibiotic therapy. While the presenting symptoms were nonspecific, the clinical features aligned with the literature—chronic painless otorrhea with hearing loss and extensive damage to the tympanic membrane [1], although the patient denied facial nerve paralysis, which is one of the pathognomonic symptoms of tuberculous otitis media [1]. The suspicion of tuberculous otomastoiditis was further confirmed by radiological findings, revealing the destruction of bilateral mastoid air cells opacity and the left ossicles, erosion of the temporal bone with bony sequester, enlargement of cervical lymph nodes, and abscesses with fistula formation indicative of advanced tuberculous osteomastoiditis [8]. Other radiological findings, which may be seen in tuberculous otomastoiditis but was not observed in our patient, include sclerosis of the mastoid cortex, mucosal thickening of the bony external auditory canal (EAC), and soft tissue extension to the EAC [8]. In addition, CT scans and MRI also showed erosion of the clivus and the C1-C2 vertebrae, stenosis of the cervical spinal cord, abscess of the retropharyngeal space extending to the spinal canal, and paravertebral abscess with posterior epidural involvement, collectively suggestive of tuberculous spondylitis [9]. MRI is considered the modality of choice in spinal infection due to its’ high sensitivity and specificity in visualizing soft tissues. In tuberculous spondylitis, MRI may reveal abnormal paraspinal findings, a thin and smooth abscess wall, soft tissue, or intraosseous abscess, and hyperintense signal on T2-weighted images, typically involving multiple vertebral bodies [9]. However, while the clinical and radiological findings were consistent with otomastoiditis and spondylitis, the diagnosis of the patient was complicated by the false-negative staining, culture, and molecular testing. This may be explained by the low mycobacterial counts in extrapulmonary specimens, in addition to potential secondary infection [10]. Notably, acid-fast staining has a very low sensitivity in detecting tuberculous otitis media with only 20% of detection rate [11]. Nonetheless, the histopathological findings of caseous necrosis and datia Langhans cells confirmed the diagnosis of tuberculosis infection.

The nonspecific symptoms of tuberculous otomastoiditis may mimic those of complicated chronic bacterial otitis media, as observed in our patient who was initially diagnosed with chronic otitis media with neck abscesses. While the clinical presentation of patients with tuberculous otitis media and chronic bacterial otitis media can be similar, tuberculous otitis media is more commonly associated with painless otorrhea, extensive, and/or multiple perforation of the tympanic membranes, facial nerve palsy, and sensorineural hearing loss [12]. Additionally, radiographic findings may reveal denser and more widespread mastoid sclerosis, persistent mucosal thickening of the EAC, and larger soft tissue masses in the middle ear in tuberculous otomastoiditis compared to other types of chronic otitis media [8].

Tuberculous otomastoiditis may arise from various routes including the aspiration of mucus through the Eustachian tube, direct extension from the nasopharynx, hematogenous dissemination from other tuberculous foci, and direct implantation through the EAC and tympanic membrane perforation, with the aspiration of mucus and direct extension from the nasopharynx being more common [8,13]. In this patient, the presence of bilateral tuberculous otomastoiditis suggested that the primary infection occurred in the cervical spine and subsequently spread directly to the paravertebral organs, including the retropharyngeal space, and to both middle ears, resulting in the destruction of mastoid air cells and the temporal bone [11].

In summary, tuberculous otomastoiditis should always be suspected in patients with chronic otitis media refractory to antibiotic therapy. Radiological, microbiological, and histopathological examinations may aid in the diagnosis of tuberculous otomastoiditis. The identification of bilateral otomastoiditis and involvement of other organs, such as spondylitis, should trigger further investigation into the potential presence of tuberculosis infection in order to halt disease progression and prevent the onset of debilitating complications.

## Data availability statement

The data used for this case report are available from the corresponding author upon reasonable request.

## Patient consent

Written consent was obtained from the patient and his guardian for this case report.
